# Supplementary material for: Isolation and Characterization of Tissue Resident CD29-Positive Progenitor Cells in Livestock to Generate a Three-Dimensional Meat Bud
Source: Cells. 2021 Sep 21;10(9):2499. doi: 10.3390/cells10092499 (PMC8466368; doi:10.3390/cells10092499)
Supplement: Supplementary file 1 [file cells-10-02499-s001.zip › cells-1345866 Sup_Figures&Table-update-final .pdf]

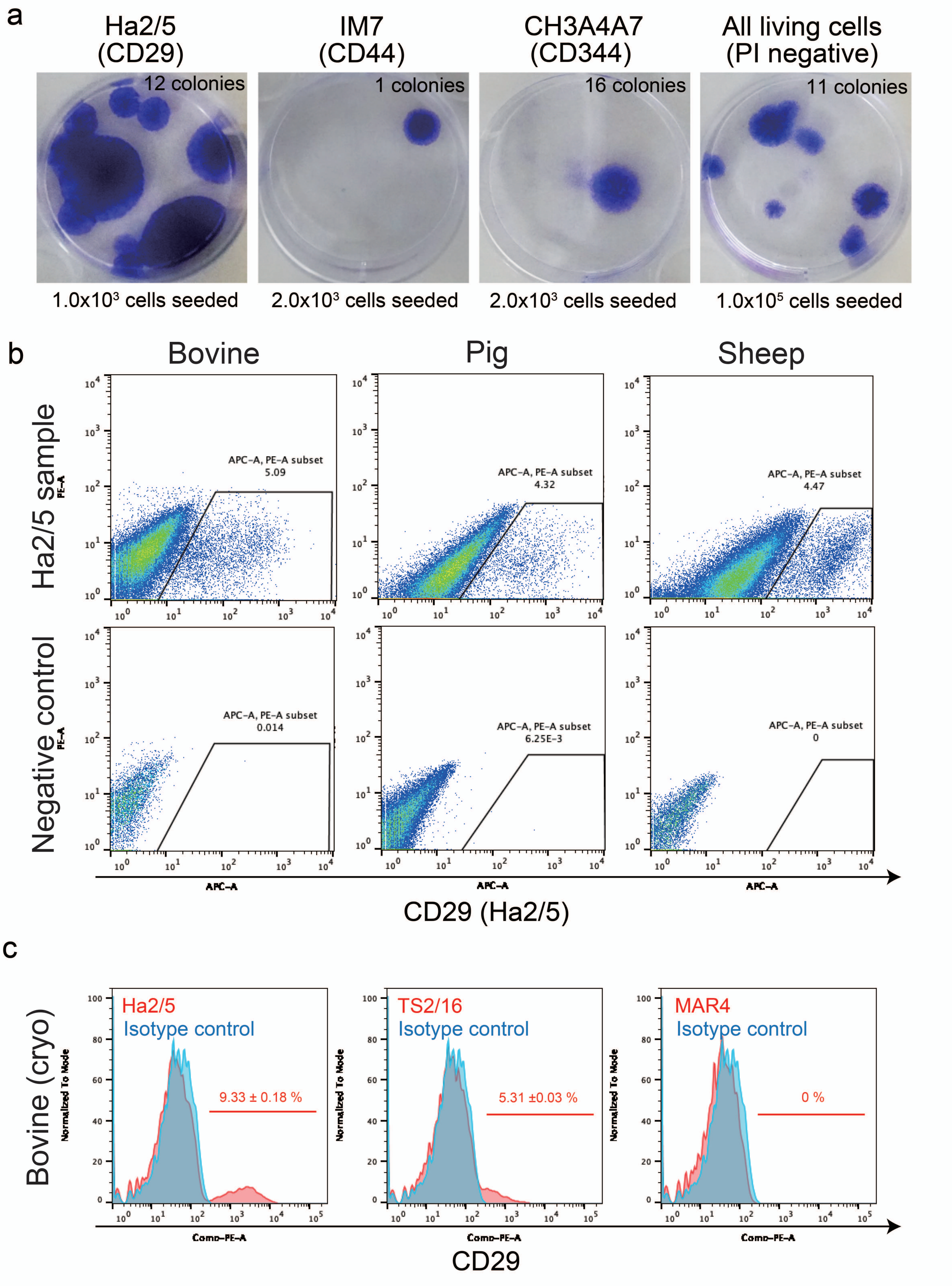

Supplementary Figure S1

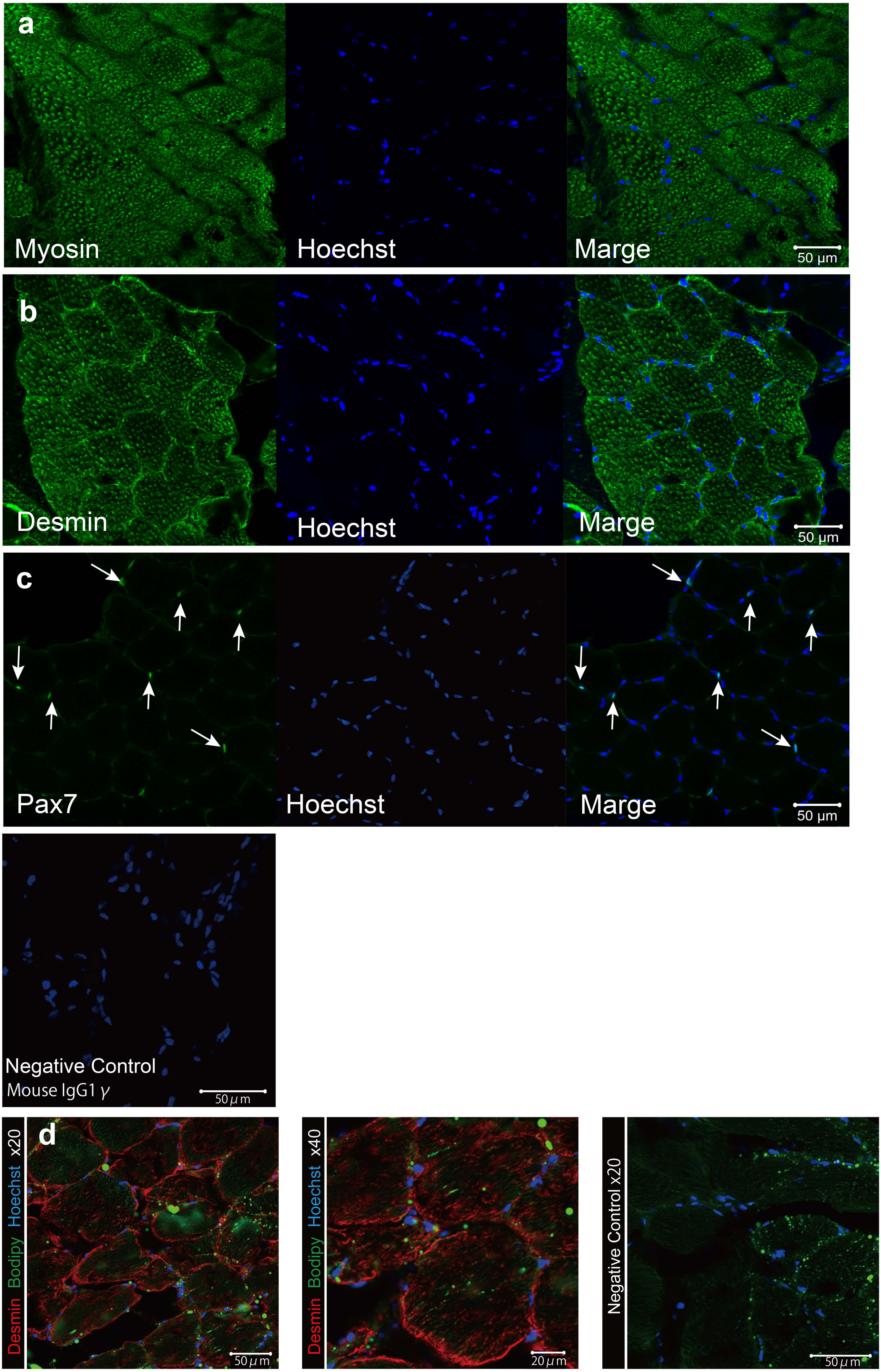

Supplementary Figure S2

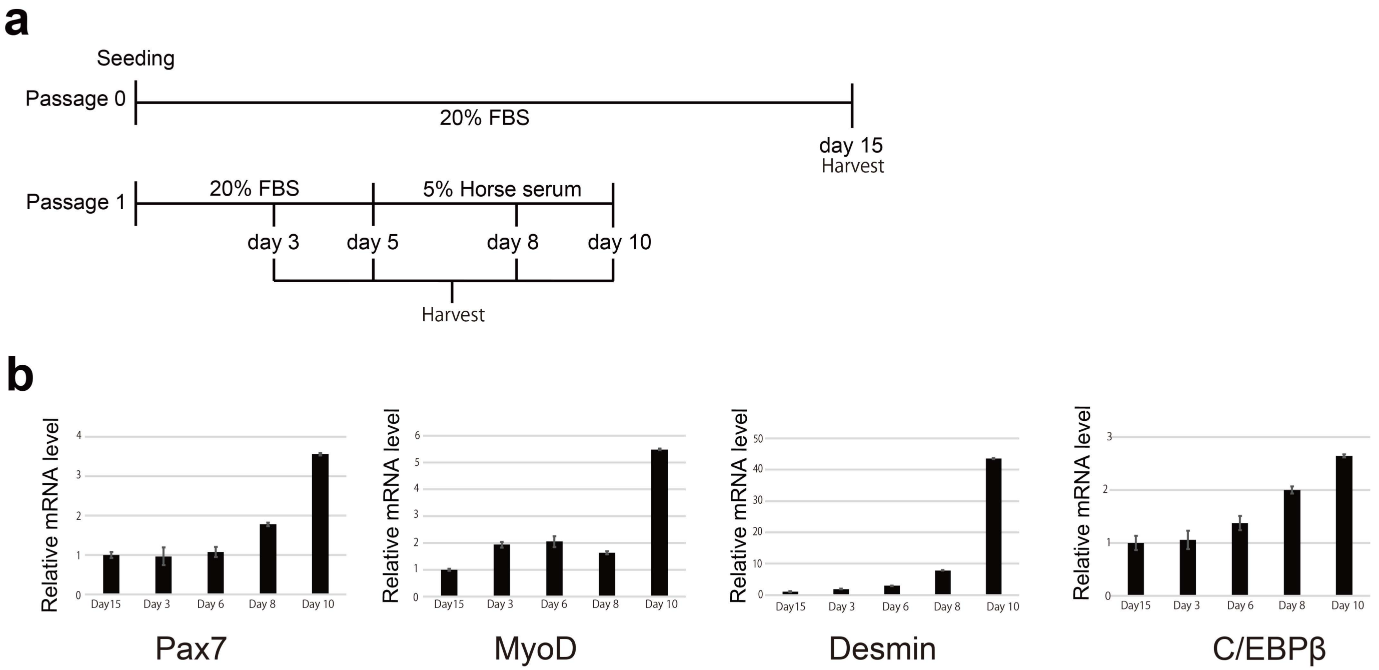

Supplementary Figure S3

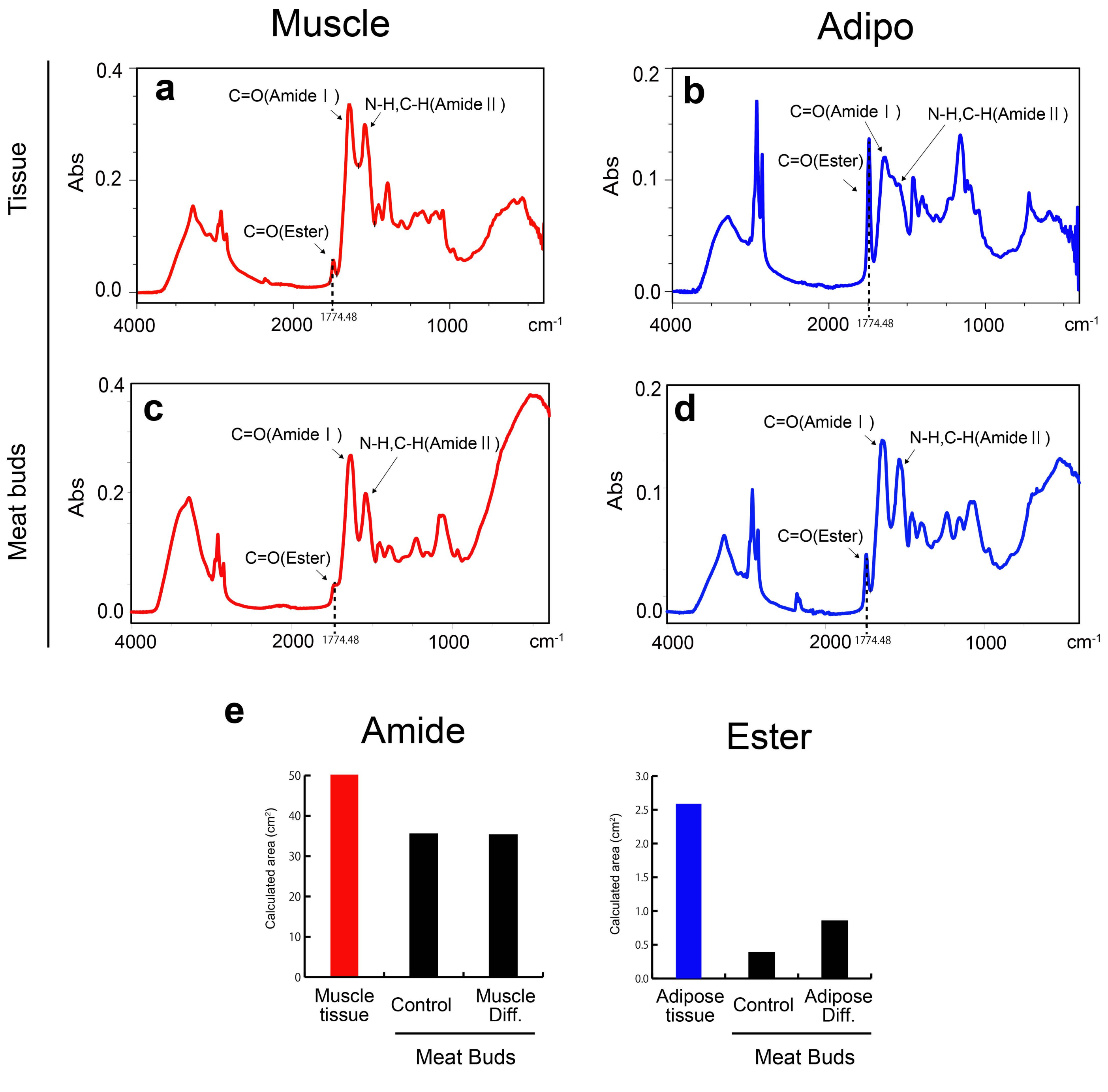

Supplementary Figure S4

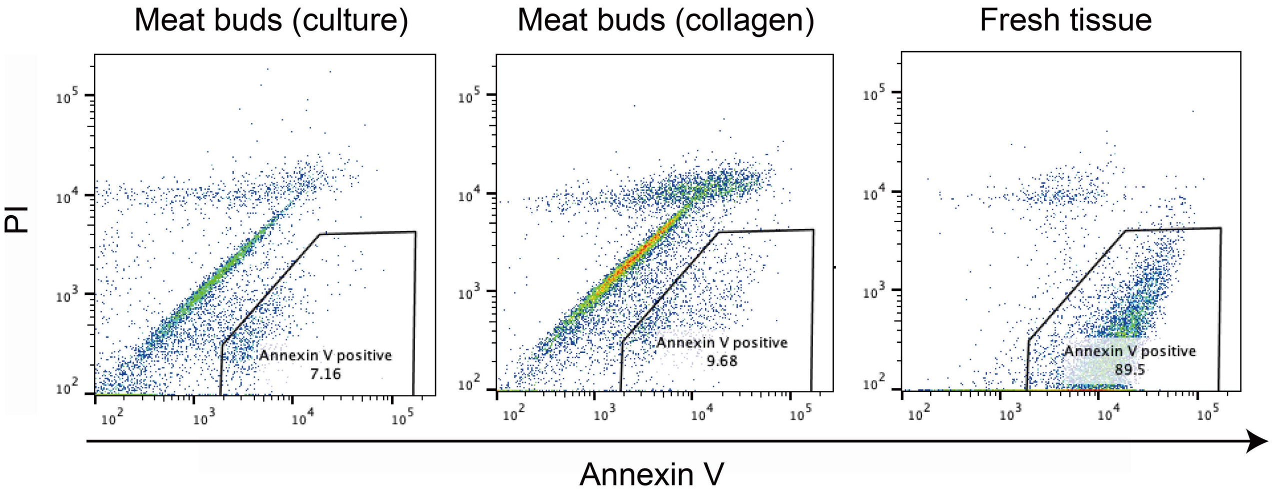

Supplementary Figure S5

Supplementary Table S1: Antibodies used for cell surface marker screening

Reactivity	Antigen	Clone
Human	CD3	SK7
	CD3	UCHT1
	CD4	SK3
	CD8	RPA-T8
	CD9	M-L13
	CD11a	G43-25B
	CD11b	2LPM19c
	CD11b	ICRF44
	CD11b	D12
	CD11c	S-HCL-3
	CD17	G035
	CD18	6.7
	CD19	HD37
	CD25	2A3
	CD29	MAR4
	CD29	TS2/16
	CD31	WM59
	CD31	MBC78.2
	CD31	REA730
	CD34	581
	CD41a	HIP8
	CD42b	HIP1
	CD44	515
	CD44	G44-26
	CD45	HI30
	CD45	REA747
	CD45	2D1
	CD48	TÜ145
	CD49a	SR84
	CD49b	12F1
	CD49c	C3II.1
	CD49d	9F10
	CD49e	IIA1
	CD49f	GoH3
	CD51/61	23C6
	CD52	4C8
	CD55	JS11
	CD56	B159
	CD56	MEM188
	CD56	NCAM16.2
	CD61	VI-PL2
	CD62E	68-5H11
	CD62L	DREG-56
	CD63	H5C6
	CD65	VIM8
	CD68	Y1/82A
	CD73	REA804
	CD73	AD2
	CD81	JS-81
	CD82	REA221
	CD82	ASL-24
	CD90	5.00E+10
	CD101	V7.1
	CD102	CBR-1C2/2
	CD103	Ber-ACT8
	CD104	439-9B
	CD105	266
	CD106	STA
	CD106	51-10c9
	CD110	1.6.1
	CD111	CK41
	CD115	9-4D2-1E4

Reactivity	Antigen	Clone
Human	CD116	hGMCSFR-M1
	CD120b	hTNFR-M1
	CDw125	A14
	CD126	M5
	CD131	3D7
	CD132	AG184
	CD133	W6B3C1
	CD135	4G8
	CD136	Ztlg4
	CD138	MI15
	CD140a	aR1
	CD140a	16A1
	CD144	55-7H1
	CD146	P1H12
	CD150	A12
	CD156c	11G2
	CD157	SY/11B5
	CD160	BY55
	CD166	3A6
	CD182	6C6
	CD184	1D9
	CD194	1G1
	CD199	112509
	CD200	OX-104
	CD201	RCR-401
	CD201	RCR-252
	CD203c	NP4D6
	CD204	U23-56
	CD207	2G3
	CD208	I10-1112
	CD210a	3F9
	CD212	2.40E+06
	CD215	JM7A4
	CD218a	H44
	CD220	3B6/IR
	CD223	T47-530
	CD234	NaM185-203
	CD235a	REA175
	CD235a	GA-R2(HIR2)
	CD239	B64
	CD249	2D3/APA
	CD252	ik-1
	CD253	RIK-2
	CD266	ITME-1
	CD271	C40-1457
	CD271	ME20.4-1.H4
	CD272	JC68-540
	CD277	232-5
	CD284	TF901
	CD300c	TX45
	CD309	89106
	CD318	CUB1
	CD324	67A4
	CD325	8C11
	CD328	F023-420
	CD344	CH3A4A7
	CD354	6B1
	CXCR5	RF8B2
	E-cadherin	36/E-Cadherin
	Fibroblast	REA165
	Foxp3	259D/C7
	IgG	G18-145

Reactivity	Antigen	Clone
Human	IL-21R	17A12
	Leptin R	52263
	P-glycoprotein	17F9
	SSEA-3	MC-631
	Stro-1	STRO-1
	TLR9	eB72-1665
Mouse	CD3	17A2
	CD3e	1452c11
	CD4	RM4-C5
	CD4	GK1.5
	CD8a	53-6.7
	CD9	KMC8
	CD11a	2D7
	CD11b	M1/70
	CD11c	HL3
	CD11c	N418
	CD18	C71/16
	CD25	PC61
	CD27	LG.3A10
	CD31	MEC13.3
	CD31	390
	CD34	HM34
	CD34	RAM34
	CD36	CRF D-2712
	CD40	HM40-3
	CD41	MWReg30
	CD44	IM7
	CD45	30-F11
	CD45R	RA3-6B2
	CD48	HM48-1
	CD49a	HA31/8
	CD49b	HMALPHA2
	CD49d	R1-2
	CD49d	DATK32
	CD49e	5H10-27
	CD51	RMV-7
	CD54	3E2
	CD61	2C9.G2
	CD62L	MEL-14
	CD63	NVG-2
	CD73	TY/11.8
	CD73	TY/23
	CD81	Eat2
	CD90.2	30-H12
	CD90.2	53-2.1
	CD102	3C4
	CD103	M290
	CD105	MJ7/18
	CD106	429
	CD117	2B8
	CD120b	TR75-89
	CD126	D7715A
	CD133	315-2C11
	CD135	A2F10.1
	CD138	281-2
	CD140a	APA5
	CD144	11D4.1
	CD146	ME-9F1
	CD154	MR1
	CD169	3D6.112
	CD192	SA203G11
	CD196	29-2L17

Reactivity	Antigen	Clone
Mouse	CD200	OX-90
	CD206	C068c2
	CD210	1B1.3a
	CD284	MTS510
	CD309	AVAS12α1
	F4/80	BM8
	Gr-1	RB6-8C5
	Integrin-α7	
	p75NGF-R	MLR2
	Sca-1	D7
	Sca-1	E13-161.7
	Ter119	Ter119
Rat	CD1d	WTH2
	CD2	OX34
	CD3	G4.18
	CD4	OX-35
	CD5	OX-19
	CD6	OX-52
	CD8a	OX-8
	CD8b	341
	CD11b	WT.5
	CD11b/c	OX-42
	CD18	WT.3
	CD24	HIS50
	CD25	OX-39
	CD26	OX-61
	CD28	JJ319
	CD29	Ha2/5
	CD31	TLD-3A12
	CD32	D34-485
	CD42d	RPM.4
	CD44H	OX49
	CD45	OX-1
	CD45RA	OX-33
	Cd49b	Ha1/29
	CD49d	MRα4-1
	CD54	1A29
	CD59	TH9
	CD61	F11
	CD62L	HRL1
	CD71	OX-26
	CD80	3H5
	CD86	24F
	CD90	OX-7
	CD103	OX-62
	CD106	MR106
	CD134	OX-40
	CD161a	10/78
	CD172	OX-41
	CD314	9C11G4
	Erythroid cells	HIS49
	αβTCR	R73
	γδTCR	V65
	Granurocytes	RP-1
	RT1A	B5
	RT1B	OX-6
	RT1D	OX-17
	STRO-1	Stro-1
Sheep	CD31	CO.3E1D4
	CD45	1.11.32

	0% ≤
	1% ≤
	5% ≤
	10% ≤
